# Prediction of risk and clinical outcome of cuproptosis in lung squamous carcinoma

**DOI:** 10.1186/s12890-023-02490-9

**Published:** 2023-06-12

**Authors:** Yangyang Zhang, Jia Zhou, Hong Li, Yaobang Liu, Jinping Li

**Affiliations:** 1 Ningxia Medical University, Yinchuan, Ningxia China; 2 Ningxia Hui Autonomous Region People’s Hospital, Yinchuan, Ningxi China; 3 Department of Surgical Oncology, General Hospital of Ningxia Medical University, Yinchuan, Ningxia China

**Keywords:** Cuproptosis, Lung squamous cell carcinoma, Immune infiltration, Tumor microenvironment, Drug sensitivity

## Abstract

**Background:**

Lung squamous cell carcinoma (LUSC) is an important subtype of non-small cell lung cancer. Its special clinicopathological features and molecular background determine the limitations of its treatment. A recent study published on Science defined a newly regulatory cell death (RCD) form – cuproptosis. Which manifested as an excessive intracellular copper accumulation, mitochondrial respiration-dependent, protein acylation-mediated cell death. Different from apoptosis, pyroptosis, necroptosis, ferroptosis and other forms of regulatory cell death (RCD). The imbalance of copper homeostasis in vivo will trigger cytotoxicity and further affect the occurrence and progression of tumors. Our study is the first to predict the prognosis and immune landscape of cuproptosis-related genes (CRGs) in LUSC.

**Methods:**

The RNA-seq profiles and clinical data of LUSC patients were downloaded from TCGA and GEO databases and then combined into a novel cohort. R language packages are used to analyze and process the data, and CRGs related to the prognosis of LUSC were screened according to the differentially expressed genes (DEGs). After analyzed the tumor mutation burden (TMB), copy number variation (CNV) and CRGs interaction network. Based on CRGs and DEGs, cluster analysis was used to classify LUSC patients twice. The selected key genes were used to construct a CRGs prognostic model to further analyze the correlation between LUSC immune cell infiltration and immunity. Through the risk score and clinical factors, a more accurate nomogram was further constructed. Finally, the drug sensitivity of CRGs in LUSC was analyzed.

**Results:**

Patients with LUSC were divided into different cuproptosis subtypes and gene clusters, showing different levels of immune infiltration. The risk score showed that the high-risk group had higher tumor microenvironment score, lower tumor mutation load frequency and worse prognosis than the low-risk group. In addition, the high-risk group was more sensitive to vinorelbine, cisplatin, paclitaxel, doxorubicin, etoposide and other drugs.

**Conclusions:**

Through bioinformatics analysis, we successfully constructed a prognostic risk assessment model based on CRGs, which can not only accurately predict the prognosis of LUSC patients, but also evaluate the patient 's immune infiltration status and sensitivity to chemotherapy drugs. This model shows satisfactory predictive results and provides a reference for subsequent tumor immunotherapy.

**Supplementary Information:**

The online version contains supplementary material available at 10.1186/s12890-023-02490-9.

## Introduction

Globally, lung cancer is the cancer with the highest mortality and the second highest incidence [1]. According to histopathological types, lung cancer can be divided into small cell lung cancer and non-small cell lung cancer (NSCLC). NSCLC accounts for about 85% of all lung cancer cases and is the main pathological type of lung cancer morbidity and mortality, which is further divided into lung adenocarcinoma (LUAD), lung squamous cell carcinoma (LUSC), large cell lung cancer and other subtypes [2]. LUSC accounts for about 25% to 30% of NSCLC cases and is the second most common subtype after LUAD [3]. Its unique clinicopathological features, including the fact that the tumors are mostly located in the central site, and most of the patients are diagnosed with late stage, advanced age and multiple complications, which determines the limitations of the treatment of LUSC [4]. Although surgery is the recommended treatment for early NSCLC, more than 60% of patients are diagnosed with locally advanced (stage III, IV) or metastasis, and often lose the chance of surgery [5, 6]. For advanced LUSC, the main clinical methods are radiotherapy, chemotherapy, targeted therapy and biological therapy [7]. Platinum-containing double-drug chemotherapy is still the main treatment method for the majority of LUSC patients, and its therapeutic effect has reached a plateau. LUSC does not have a clear driver mutation gene like LUAD and does not benefit well from targeted drug therapies for driver genes such as epidermal growth factor receptor ( EGFR) and anaplastic lymphoma kinase (ALK) [8, 9]. Based on the current findings, immunotherapy is expected to prove to be a novel treatment for lung cancer that is not limited by histological subtypes [10]. It can be seen that the treatment of lung squamous cell carcinoma poses great challenges to clinical decision makers. Therefore, it is urgent to find specific biomarkers for early assessment of the prognosis of patients with lung squamous cell carcinoma, so as to further improve the treatment and prognosis of patients.

Programmed cell death is an active and orderly way of cell death controlled by genes, which is crucial to the maintenance and development of tissue homeostasis and the removal of damaged, diseased or dead cells in multicellular organisms. Its mechanisms include apoptosis, pyrodeath, autophagy, necrosis, etc. In March 2022, Science published an article describing a new form of cell death called cuproptosis. It is a new form of programmed cell death induced by the mitochondrial tricarboxylic acid cycle (TCA) that is different from the previously recognized cell death process [11]. Copper is one of the essential trace elements in the human body and a necessary auxiliary regulator of various enzyme reactions. It is also strictly regulated by copper chaperone proteins and copper transporters [12]. The content of copper in the body always maintains a dynamic balance. Excess or lack of copper can lead to cytotoxicity and eventually induce cell death through a variety of ways [13]. Abnormal copper metabolism can lead to a variety of diseases. *ATP7B* gene mutation leads to overload of copper in liver and neurons and induces Wilson's disease [14]. Menkes disease with *ATP7A* gene mutation causing loss of copper absorption and utilization [15]. Changes in copper homeostasis have been demonstrated in age-related neurodegenerative diseases such as Alzheimer's and Parkinson's disease [16, 17]. In addition, the content of copper in serum and tumor tissues of most cancer patients was found to be higher than that of healthy people, including breast cancer, lung cancer, cervical cancer, etc.. Suggesting that copper death can be used as a potential biological target for diagnosis or treatment of these diseases [18–20].

In recent years, the emergence of immunotherapy has largely changed the treatment status of driver gene negative advanced NSCLC. Among them, immune checkpoint inhibitor therapy targeting the immune escape mechanism of tumor cells has developed rapidly in LUSC, represented by anti-programmed death receptor/ligand 1 (PD-1/PD-L1) and anti-cytotoxic T lymphocyte antigen 4 (CTLA-4) [21]. Multiple studies such as RATIONALE-307, KEYNOTE-407, and CheckMate-017 have found that immunotherapy alone or in combination with chemotherapy can significantly improve the prognosis of patients with advanced LUSC [22–24].

In our study, the relationship between cuproptosis and tumor microenvironment of LUSC was first explored. Based on the analysis of high-throughput sequencing data, the expression of cuproptosis biomarkers in 730 LUSC patients was studied. A clinical prognostic model based on cuproptosis model was established and its ability to predict the prognosis of LUSC was verified. The mechanism of differentially expressed genes related to cuproptosis in the occurrence and development of LUSC was analyzed by functional enrichment analysis. R language was used to evaluate the correlation between risk score and clinicopathological features, immune cell infiltration and chemotherapy sensitivity. It was speculated that cuproptosis was closely related to the immune function of LUSC, providing new ideas and methods for individualized immunotherapy and drug therapy of LUSC.

## Method

### Data source and preprocessing

RNA sequencing data ( including treatment information, somatic mutation data and CNV data files) and clinical data ( phenotype and survival data) were obtained from The Cancer Genome Atlas ( TCGA) LUSC cohort of the Genomic Data Commons ( GDC), which contained information on a total of 49 normal individuals and 502 patients with LUSC. The database was accessed at https://portal.gdc.cancer.gov/. Additionally, gene expression array GSE157010 containing 235 LUSC patients and their corresponding clinical data was retrieved as a supplementary data set from the Gene Expression Omnibus (GEO) ( https://www.ncbi.nlm.nih.gov/geo/). In order to reduce potential bias, we excluded patients who lacked clinical and survival data. After that, a total of 730 LUSC cases were included for analysis after combining the two data sets obtained.

### Expression of cuproptosis related genes (CRGs)

We reviewed the published literature and summarized cuproptosis genes, and obtained 19 genes [11, 25–29]. The transcription data downloaded in the TCGA dataset was presented in the form of millions of fragments per kilobase (FPKM) values. The expression levels of CRGs were extracted from 49 normal tissues and 502 tumor tissues by “Limma” package in R language.

### Detection of somatic CNV and its mutation

The LUSC somatic mutation data ( SNV) downloaded from GDC were analyzed using the R package “mafTools” and reflected by a waterfall plot. The R package “igraph” is used to show common frequent mutations, tumor mutation load (TMB), and non-synonymous somatic mutations shared by different variants, as well as to construct a network diagram of interactions between CRGs. Subsequently, 10 CRGs associated with prognosis of LUSC were screened out by univariate cox analysis with *p* < 0.05 as the threshold.

### Establishment of CRGS genotyping

According to survival analysis, the R language “ConsenusClusterPlus” package was used to conduct unsupervised consensus clustering on the genes with significant survival significance and *P* < 0.05. The optimal K value is determined according to the cumulative distribution function and incremental area value. When the parameter K = 2, the stability between subtypes is the best. Kaplan–Meier (KM) survival analysis was used to compare the overall survival (OS) of the groups with R language “survival” and “surviminer” packages. Then ggplot2 software was used for principal component analysis. In addition, the R language “pheatmap” package was used to create heat maps that visualized clinical information (age, sex, tumor stage, survival time) and differences in gene expression between groups.

### Functional enrichment analysis

The “GSVA” package was used to analyze gene set variation in biological pathways of two LUSC subtypes. Single sample gene set enrichment analysis (ssGSEA) was used to compare enrichment scores of immunocyte infiltration abundance between the two subtypes. Followed by using R language “limma” package with adjusted | FoldChange |> 1.5 and *P* < 0.05 to screen the differentially expressed genes between different subtypes. In addition, R package “ClusterProfiler” (FDR < 0.05) was used for GO (Gene ontology) and KEGG (Kyoto Encyclopedia of Genes and Genomes) analysis to identify the enrichment pathway.

### Further typing of differentially expressed genes (DEGs)

The differentially expressed genes with *p* < 0.05 were screened out by univariate Cox regression analysis. According to the expression of differentially expressed genes, the samples were analyzed by secondary unsupervised consensus clustering analysis with the clustering parameter K = 3, and the clustering heat map was drawn to reflect the relationship between DEGs expression, clinical features and CRG classification. Subsequently, survival analysis was used to compare the differences in OS among different subtypes, and the expression of CRGs among different subtypes was compared with the box diagram.

### Construction and verification of prognostic model

R software “caret” package was used to divide 730 samples into training set and test set in a ratio of 1:1, and the output condition was ((*p*Value < 0.01) & (roc$AUC[2] > 0.65) & (*p*ValueTest < 0.05) & (rocTest$AUC[2] > 0.6)). The least absolute shrinkage and selection operator (LASSO) regression was performed using the R software “glmnet” package to minimize the risk of overfitting. Analyzed the trajectory of individual variables and built features with tenfold cross-validation to select minimum and optimal penalty criteria. Multiple COX analysis was used to screen candidate genes and construct a risk model in training set:

$$\mathrm{Risk Score}=\sum_{i=1}^{n}(\mathrm{Coefi }*\mathrm{ Expi})$$

The sample was divided into low-risk and high-risk subgroups based on the median risk score. The R software “ggplot2”, “ggalluvial” and “dply” packages were used to generate a Sankey map to visualize the correspondence between the high-low risk groups, different subtypes and prognosis. The box plots were used to compare the differences in risk scores among different CRG subtypes and gene subtypes, as well as the differences in CRGs expression between the high and low risk groups.

### Construction of normograph

According to the risk score, the plot command was used to draw the patient survival curve and survival state scatter plot, and the pheatmap package was used to draw the candidate gene expression heatmap. The R language “RMS” package was used to construct a normogram to predict the overall survival rate based on clinically significant features and CRG scores. In addition, model calibration was performed to compare the predicted survival results with the actual occurrence of survival over one-year, three-year and five-year periods, showing that the circuit diagram conforms to the ideal model. The Kaplan–Meier method was used to show the prognostic performance of the risk score model in the training group and the test group. R package “TimeROC” was used to draw the time-dependent ROC curve. By comparing the area under the ROC curve of one-year, three-year and five-year overall survival rate over time, the accuracy of normograph in predicting survival rate was also verified.

### Characteristic evaluation of immune cell infiltration and immune microenvironment

Spearman rank correlation test was used to analyze the correlation between risk score and immune cells, and *P* < 0.05 was considered as the significant level. The “reshape2” package was used to analyze the correlations between two candidate genes and risk scores of different immune cells. In order to evaluate and compare the tumor immune microenvironment in the high-risk and low-risk groups, the “estimate” R package was used to calculate the immune score, matrix score and estimated score of each LUSC sample. The “Maftools” package was used to analyze the tumor mutation load of each sample in the high and low risk groups, and the relationship between CRG risk score and tumor stem cells was also observed.

### Sensitivity analysis of chemotherapy drugs

Finally, pRophetic software was used to calculate the half maximal inhibitory concentration (IC50) of chemotherapeutic drugs and their dependence. Wilcoxon symbolic rank test was used to compare the IC50 differences of commonly used anti-tumor drugs in the high and low risk groups. The box plot was presented using R package “ggplot2”.

### Statistical analysis

All statistical analyses were performed on R studio using the R programming language (Version 4.2.1). Kaplan–Meier analysis was used to generate survival curves for each subgroup in the data set. Univariate and multivariate COX regression analyses were used to investigate the correlation between risk score and different clinical factors. ROC and AUC curve were used to determine the accuracy of risk model prediction. Wilcoxon rank sum test was used to compare the differences of quantitative data between the two groups. Spearman analysis was used to calculate the correlation coefficient. *P* < 0.05 was considered statistically significant.

## Result

### Identification of CRGs in LUSC

**Fig. 1:**
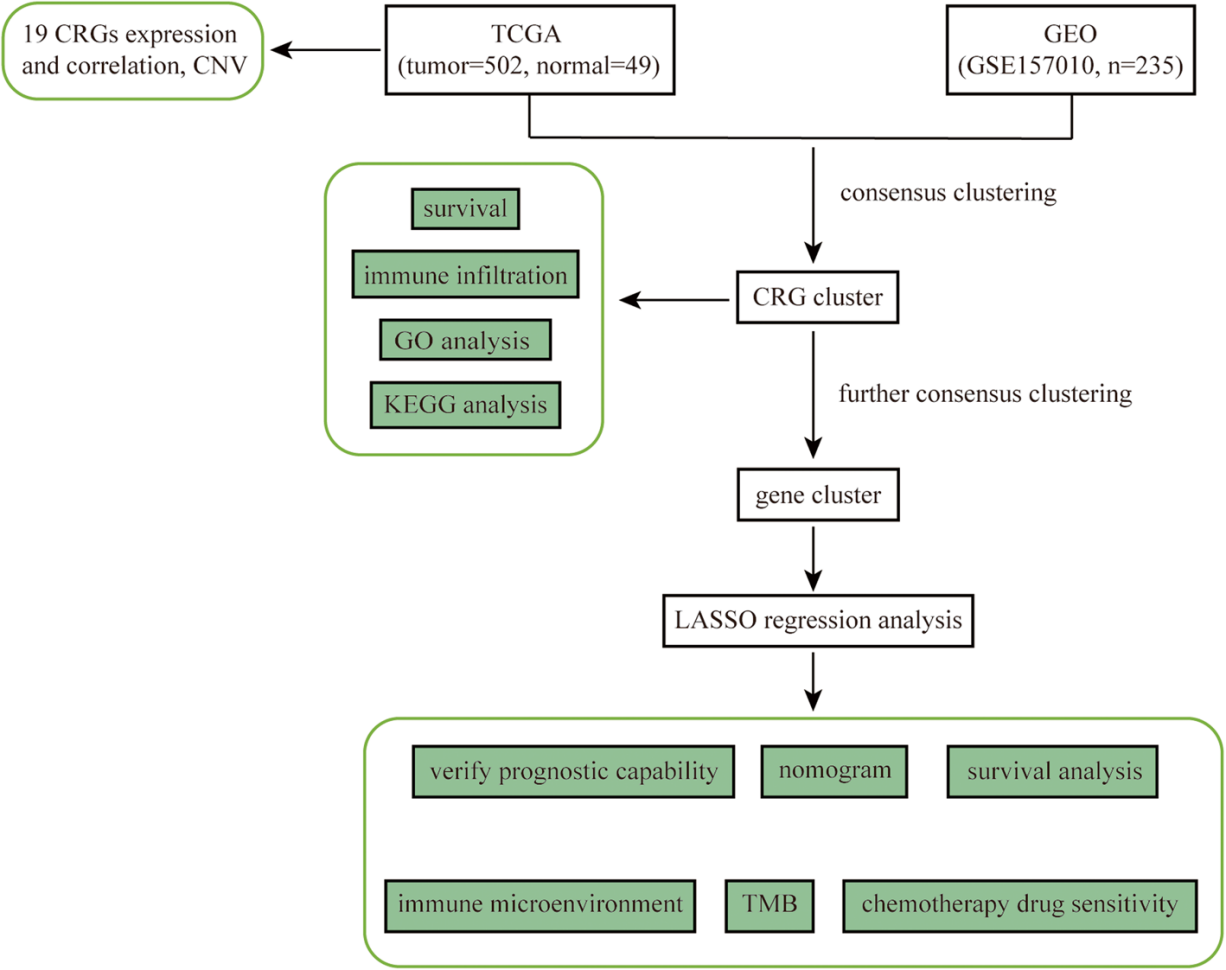
The workflow of this study

**Fig. 2:**
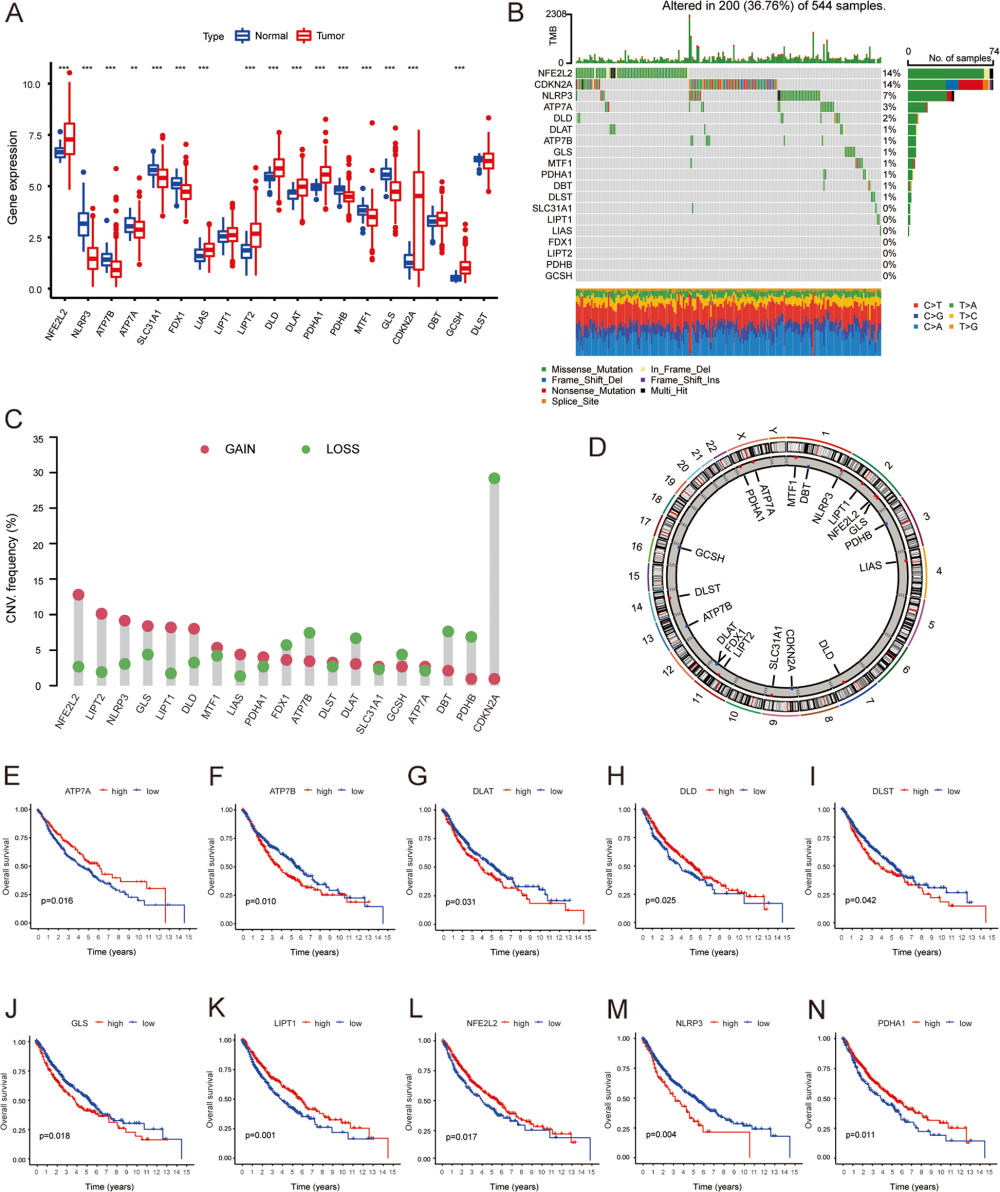
Multiomics analysis of CRGs in TCGA-LUSC. **A** Expression levels of 19 CRGs in normal lung tissue and LUSC tissue. **B** Waterfall of somatic mutation data. **C** CNV mutation sites of CRGs on 23 chromosomes. **D** Distribution of CNV gain, loss, and non-CNV in CRGs. **E**-**N** Associations between 10 CRGs and overall survival (*p* < 0.05)

The workflow of this study is illustrated in Fig. 1. The expression levels of 19 CRGs (Supplementary Table S1) were analyzed in the lung tissues of 502 LUSC patients and 49 healthy people downloaded from the TCGA dataset. It was found that except *LIPT1, DBT* and *DLST*, the expression of other 16 CRGs in LUSC tissues was significantly different from that in normal tissues (Fig. 2A). Meanwhile, gene mutation analysis showed that 200 out of 544 samples (36.76%) had CRGs mutations, among which NFE2L2 had the highest mutation rate (Fig. 2B). In LUSC samples, *NFE2L2, LIPT1, NLRP3, GLS, LIPT2, DLD, MTF, PDHA1* and *LIAS* were amplified in most patients, while *FDX1, ATP7B, DLAT, GCSH, DBT, PDHB* and *CDKN2A* were absent (Fig. 2C). The locations of these 19 CRGs on the chromosomal rcircos are shown in Fig. 2D. In order to expand the sample size and increase the reliability of the data, we added 235 LUSC samples downloaded from the GEO database and plotted the overall survival (OS) curves for 10 CRGs (Fig. 2E-N; Supplementary Table S2). Table 1 shows the clinical characteristics of patients with LUSC in TCGA and GEO databases. By studying the effect of gene expression patterns on the overall survival rate of LUSC, it was found that patients with high expression of *ATP7B, DLAT, DLST, GLS* and *NLRP3* had poor prognosis, while patients with high expression of *ATP7A, DLD, LIPT1, NFE2L2* and *PDHA1* had improved prognosis. This finding suggests that variations in CNV can influence the expression of CRGs, which are associated with the prognosis of LUSC patients, and that CRGs may serve as therapeutic targets or predictive biomarkers for LUSC.

**Table 1:** The clinic character of LUSC patients from TCGA and GEO databases

Clinic character	TCGA	GEO (GSE157010)
**Age(Year)**		
< 65	170	79
≥ 65	325	156
**Gender**		
male	366	153
female	129	82
**Stage**		
T1	110	63
T2	291	139
T3	70	31
T4	24	0
unknow	0	2
**Survival Time (Year)**		
< 1	137	28
≥ 1, < 3	210	57
≥ 3, < 5	63	45
≥ 5	82	105
unknow	3	0
**Status**		
Live	216	116
Dead	279	119

### Establishment of cuproptosis model of LUSC

**Fig. 3:**
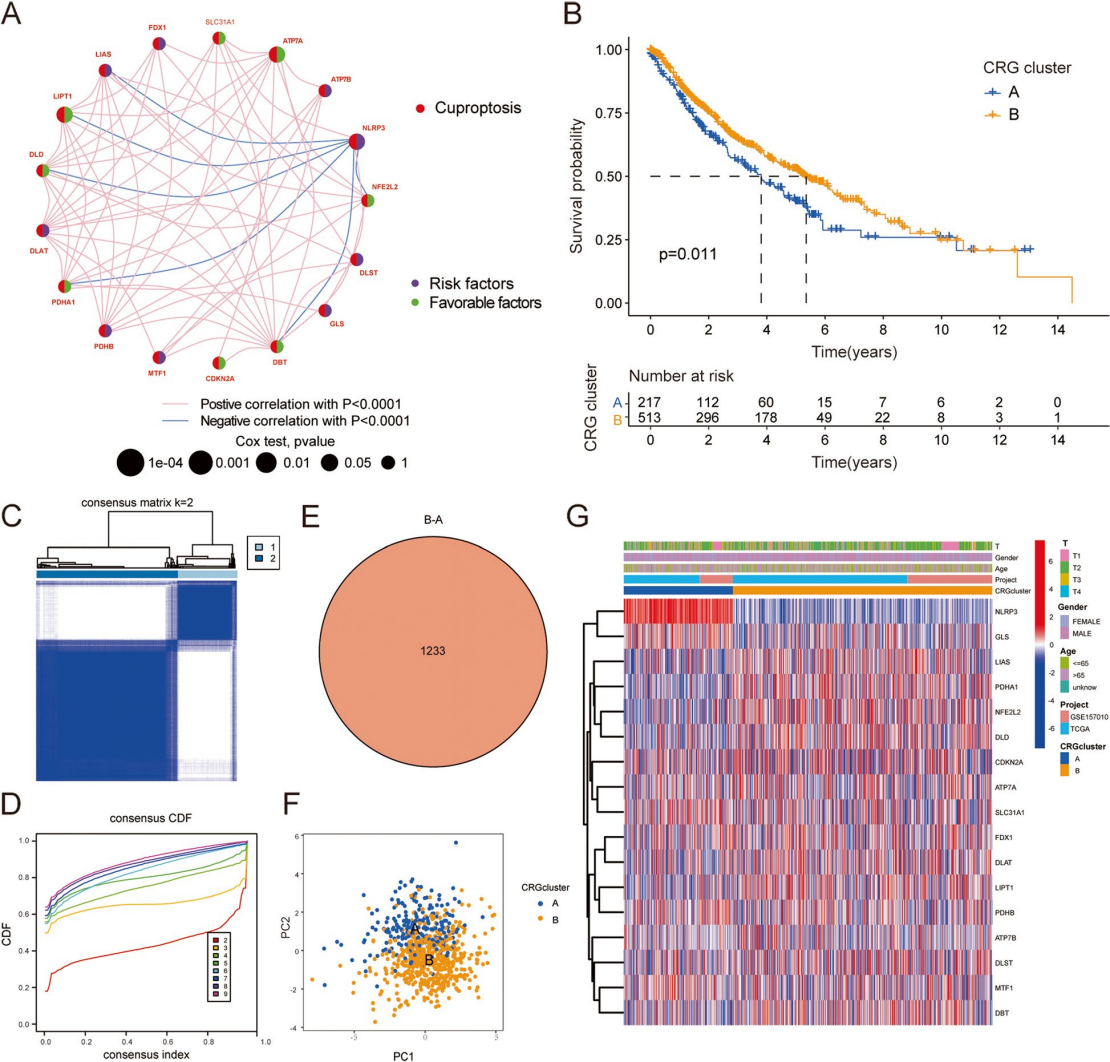
Biological and clinicopathological characteristics of two CRGs subtypes. **A** Interactions between CRGs in LUSC (red and blue strings indicate positive and negative correlations, respectively;The associated intensity is indicated by color shading). **B** Kaplan–Meier OS curve. **C**, **D** Consensus Cluster analysis of CRGs (k = 2). **E**, **F** Principal component analysis of transcriptome differences between the two subtypes. **G** Heat maps showing the expression and clinicopathological features of CRGs in two subtypes

The relational network diagram shows correlations among the 19 CRGs (Fig. 3A). Consensus clustering was performed on the data set based on DEGs (input K = 2–9). When parameter K = 2, the stability between the two subtypes was the best, that is, there was the smallest inter-group difference and the largest intra-group difference (Fig. 3C, D). Subtype A included 217 patients and subtype B included 513 patients. Kaplan–Meier survival curve analysis showed that the survival prognosis of subtype B was better than that of subtype A, and there was a significant difference (*p* = 0.011) (Fig. 3B). Principal component analysis showed that the two subtypes had different transcription patterns of cuproptosis (Fig. 3E, F). In addition, heat maps showed the expression of CRGs in two subtypes and different patient clinical data, the *NLRP3* was upregulated in subtype A and downregulated in subtype B (Fig. 3G).

### Infiltration and functional enrichment analysis of different subtypes of TME

**Fig. 4:**
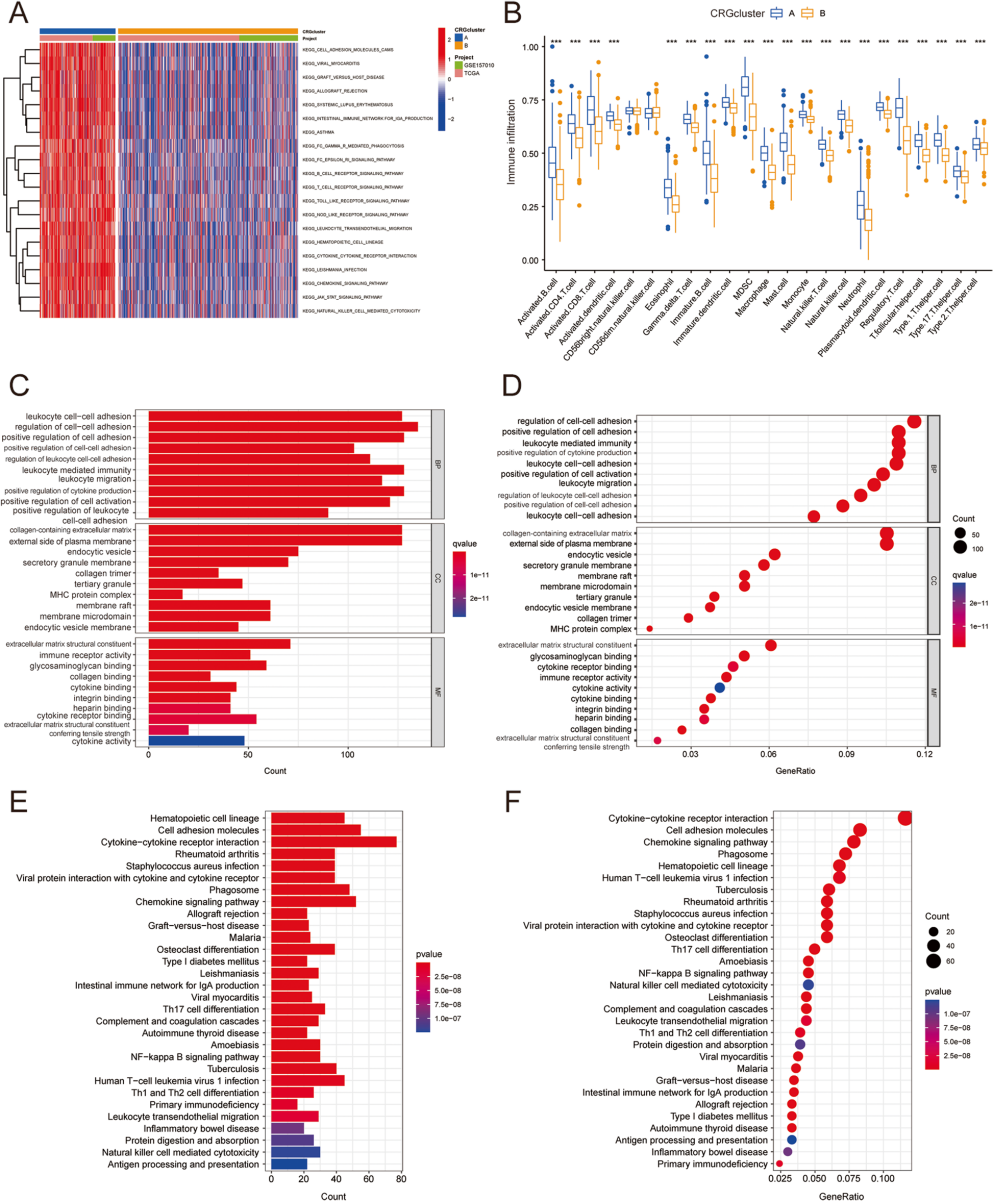
TME infiltration abundance and functional enrichment analysis of two copper dead subtypes. **A** GSVA of cell pathways associated with two copper death isoforms (red for activation, blue for inhibition). **B** Levels of immune cell infiltration between the two subtypes. **C**-**F** Functional enrichment analysis of GO and KEGG was performed for the two subtypes

According to GSVA enrichment studies, the metabolic activation pathways of subtype A are more abundant than those of subtype B (Fig. 4A). In each LUSC sample, CiberSort method was used to assess the significant difference in the number of immune cell infiltration between the two subtypes (Fig. 4B). Except that there was no significant difference in the distribution of CD56 dim natural killer cells and CD56 bright natural killer cells between the two groups, the proportion of most immune cells such as B cells, CD4T cells and CD8T cells etc. in peripheral blood of LUSC patients in group A was higher than that in group B. Subsequently, GO and KEGG functional enrichment analyses were performed to investigate the possible biological roles of these two cuproptosis subtypes. A total of 1233 DEGs were identified (Supplementary Table S3). In biological processes, genes are mainly concentrated in regulation of cell–cell adhesion, positive regulation of cell adhesion and positive regulation of cytokine production. In the cellular component, it was mainly enriched in the collagen-containing extracellular matrix, external side of plasma membrane and endocytic vesicle. In the molecule function, it was extracellular matrix structural constituent, glycosaminoglycan binding, and cytokine receptor binding (Fig. 4C, D). KEGG enrichment analysis showed that CRGs were significantly enriched in cytokine-cytokine receptor interaction, cell adhesion molecules and Chemokine signaling pathway (Fig. 4E, F).

### Determine gene subtypes using DEGs

**Fig. 5:**
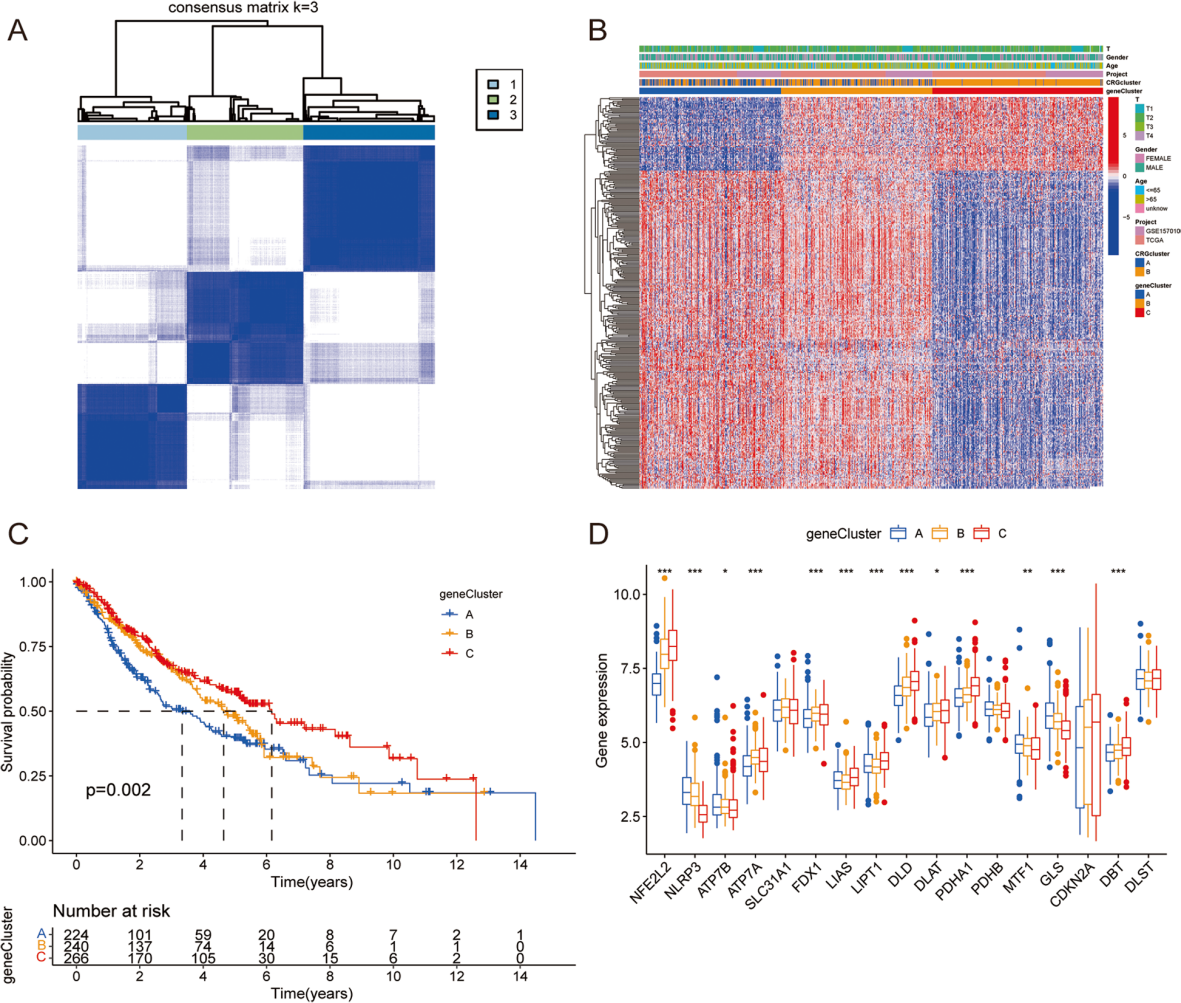
Construction of gene subtypes based on DEGs. **A** Consensus cluster analysis to identify three gene subtypes (k = 3). **B** Differences in clinical features among three gene subtypes. **C** Kaplan–Meier OS curves of three gene subtypes. **D** Differential expression of CRGs in three gene subtypes

By consensus clustering method, 730 patients with LUSC were further divided into three gene subtypes A(*n* = 224), B(*n* = 240) and C(*n* = 266) according to DEGs expression level, with parameter K = 3 (Fig. 5A). In addition, the relationship between clinical features and gene subtypes in patients with LUSC was studied (Fig. 5B). According to Kaplan–Meier curve, patients in gene subtype A had the lowest OS, while patients in gene subtype C had the highest OS, and patients in gene subtype B had an OS between subtype A and subtype C (*p* = 0.002) (Fig. 5C). It suggested that CRGs could be used as a bioinformatics tool with prognostic value in clinical practice. The expressions of CRGs of the three gene subtypes were significantly different except *SLC31A1, PDHB, CDKN2A* and *DLST* (Fig. 5D).

### Construction of risk scoring model

**Fig. 6:**
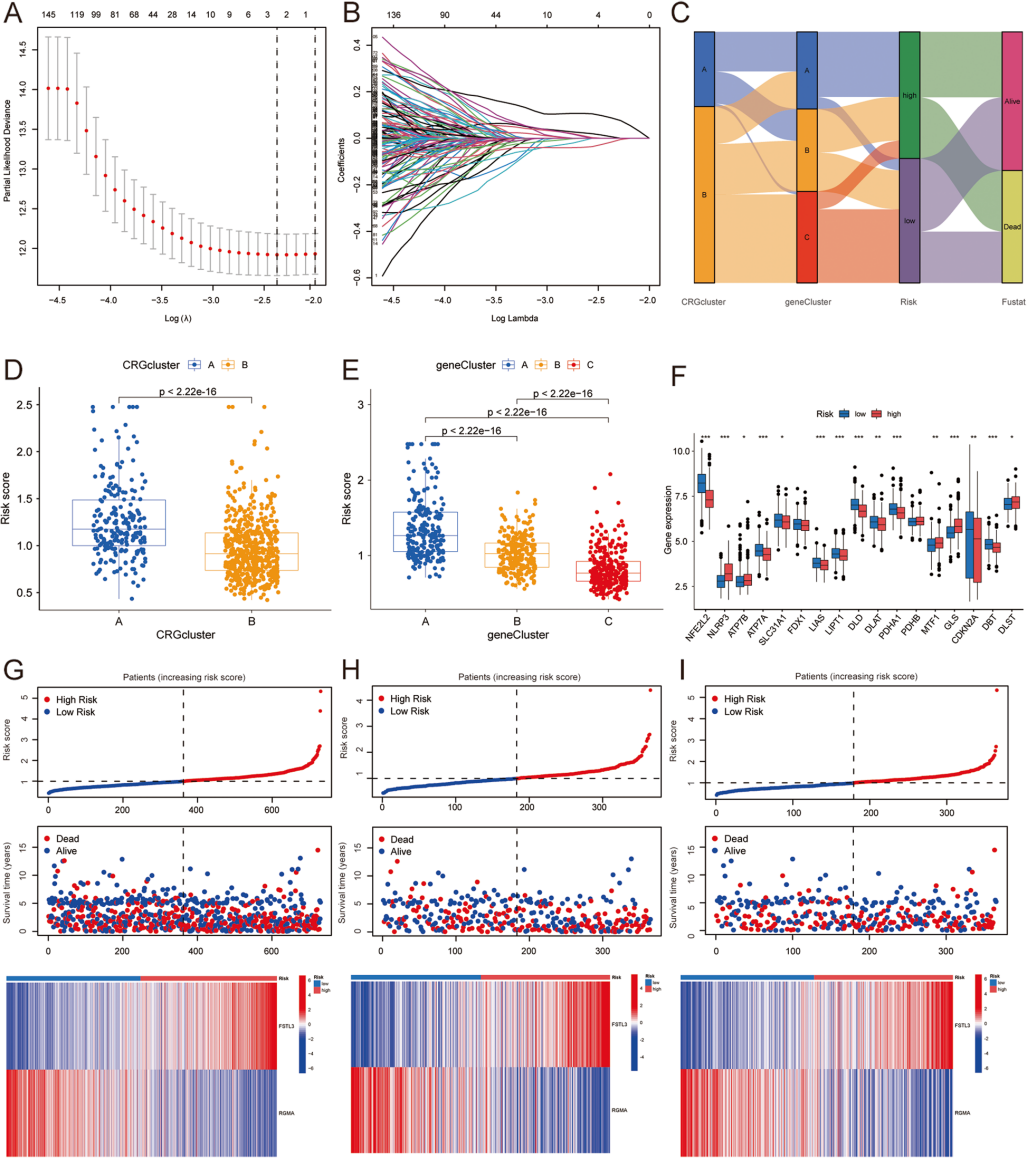
Two key genes were used to construct a risk model. **A**, **B** The risk model was constructed by Lasso regression and tenfold cross validation. **C** Correlation of subtype distribution, CRG score and survival prognosis. **D** Differences in risk scores among different cuproptosis subtypes. **E** Differences in risk scores among different gene subtypes. **F** Differences in CRGs expression in high—and low-risk groups. **G**-**I** Distribution of patient survival status and risk score in the overall sample, training set, and test set

Patients were randomly assigned to the training group (*n* = 365) and the test group (*n* = 365) at a ratio of 1: 1 (Supplementary Tables S4, S5). Lasso-Cox regression analysis and cross validation were performed based on subtype-related DEGs (Fig. 6A, B). Subsequently, two prognostic genes were screened out by multivariate cox analysis to establish a risk scoring model. The risk score of patients with lung squamous cell carcinoma = 0.2380 * FSTL3 + (-0.1532) * RGMA (Supplementary Table S6). Patients were divided into low-risk group and high-risk group according to the median risk score. The Sankey plot showed the corresponding relationship between the prognosis of different subtypes and the CRG score (Fig. 6C). There were statistically significant differences in risk scores among different cuproptosis subtypes (*p* < 0.05), and CRG score of subtype A were much higher than that of subtype B (Fig. 6D). The distribution of risk scores of the three gene subtypes was also statistically significant (*p* < 0.05), and the CRG scores were ranked as A, B, C from high to low (Fig. 6E). There were also significant differences in the expression of CRGs in high-low risk groups (Fig. 6F), suggesting that the expression of CRGs can be used to evaluate the risk of LUSC. The survival status and risk scores of patients were different between the training set and the test set. The heat map showed the expression levels of two candidate genes in the high-risk group and the low-risk group (Fig. 6G-I). The results showed that FSTL3 was highly expressed in the high-risk group and RGMA was highly expressed in the low-risk group, suggesting the practicability of CRGs in patients with LUSC.

### Influence of CRGs on prognosis of LUSC patients

**Fig. 7:**
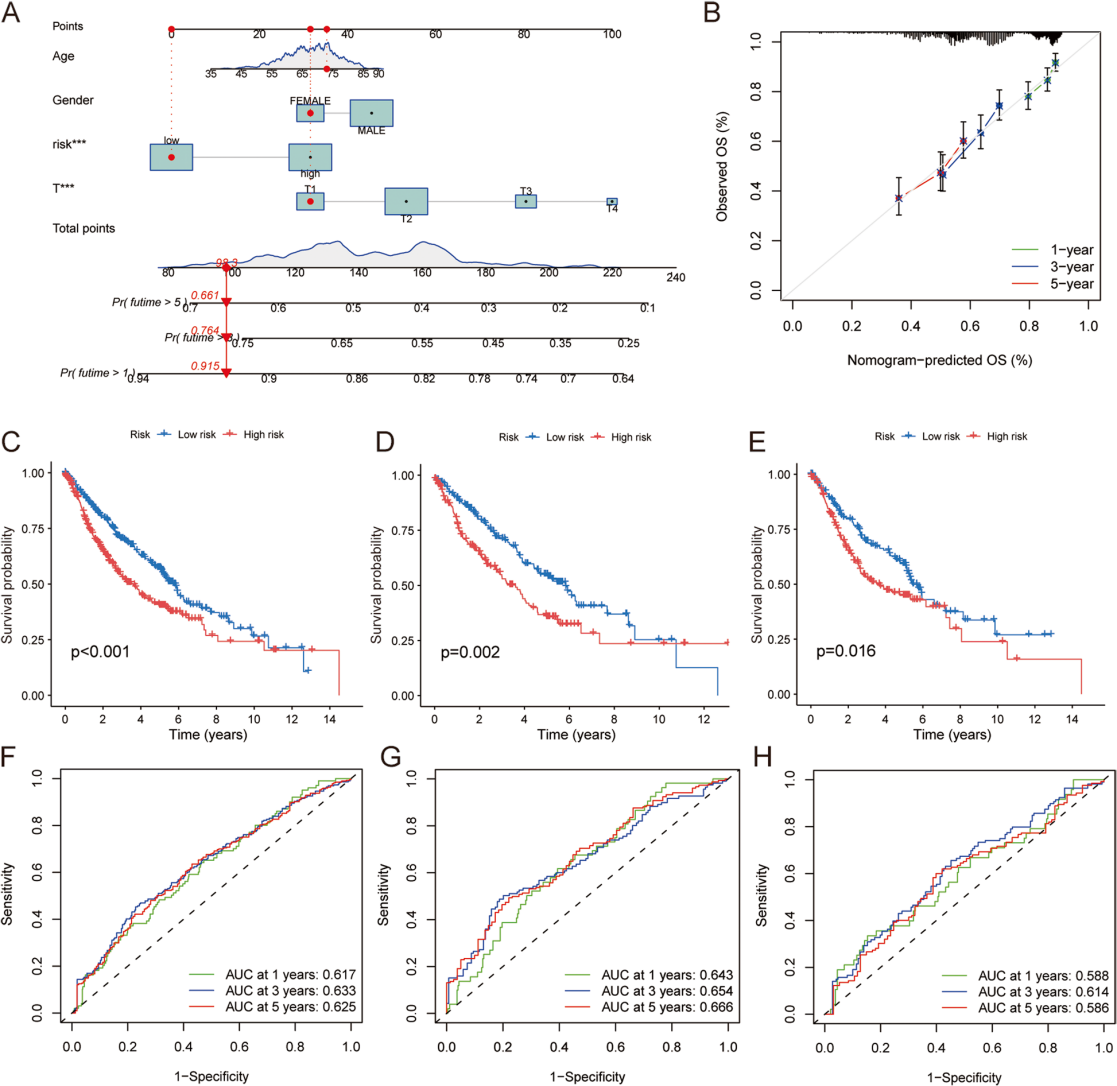
Construction of Normograph and verification of prediction effect. **A** Normograph of 1-year, 3-year and 5-year survival rates for patients with LUSC. **B** Calibration curve of Normograph. **C**-**E** KM curves based on Normograph score, population sample, training set and test set. **F**–**H** ROC curve and area under curve (AUC) of population sample, training set and test set were used to predict 1-year, 3-year and 5-year survival in the cohort respectively, once again verifying the accuracy of Normograph prediction

Using the collected data, we used the RMS program to create a nomogram to predict the prognosis of LUSC patients (Fig. 7A). The calibration nomogram predicted results are in good agreement (Fig. 7B). KM curve analysis showed that the prognosis of the low-risk group was better than that of the high-risk group in the overall sample, training set and test set (Fig. 7C-E). ROC curve analysis showed that the constructed nomogram had high accuracy in predicting patient survival (Fig. 7F–H). Taken together, the nomogram model of risk score and clinicopathological features can accurately predict the survival of LUSC patients.

### Relationship between risk score and tumor immune microenvironment

**Fig. 8:**
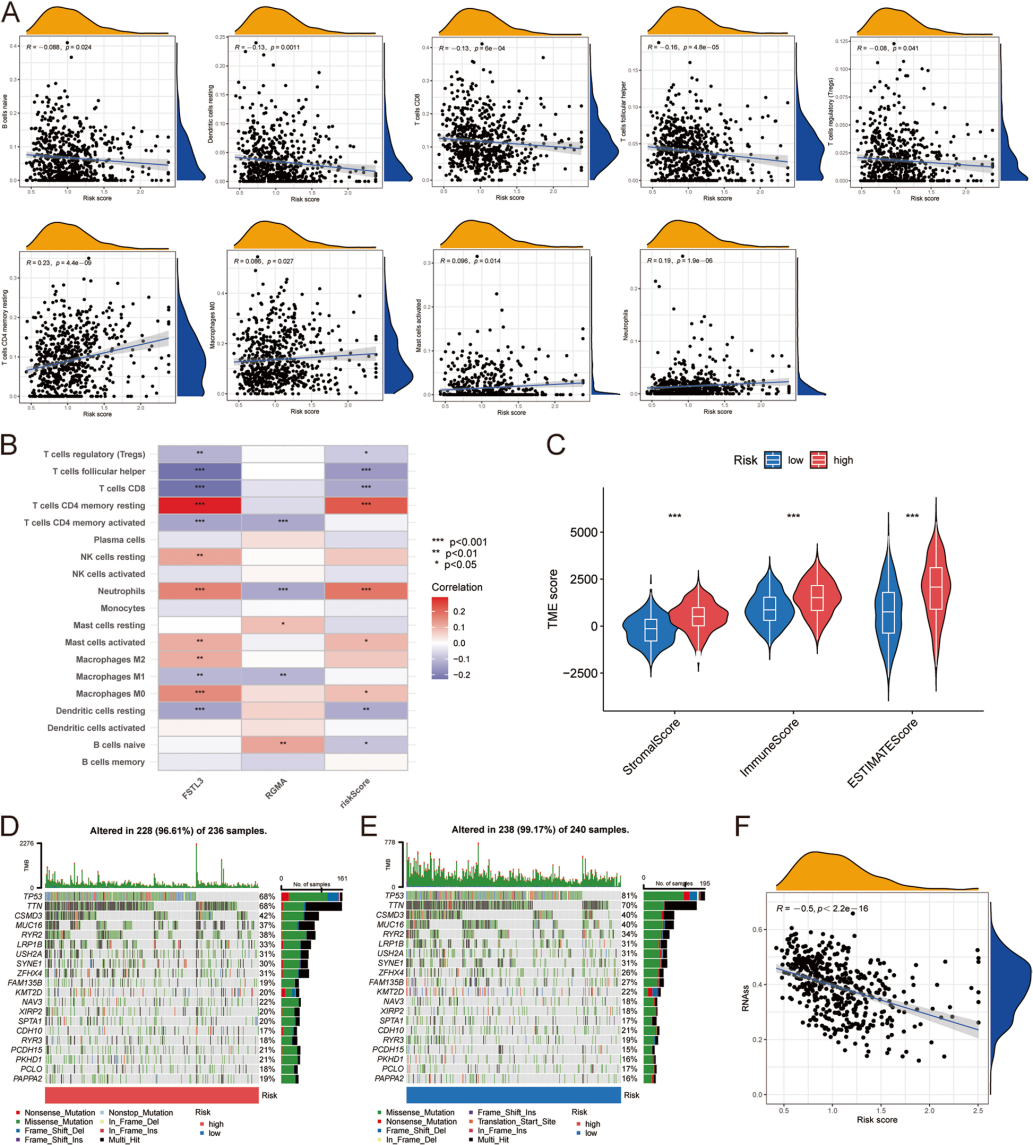
Different characteristics were exhibited between high- risk and low-risk groups. **A** Association between immune cell type and risk score. **B** There was a correlation between two candidate genes of different immune cells and risk scores. **C** TME scores between high-risk and low-risk groups. **D**, **E** Waterfall characteristics of somatic mutations in high—and low-risk groups. Each column represents a patient, the number on the right represents the mutation frequency of each gene, and the bar graph above shows the TMB. The proportions of each variation type are shown in the bar chart on the right. **F** Tumor stem cells were negatively associated with risk scores

Tumor microenvironment refers to the ecosystem surrounding tumor cells that is closely related to the occurrence, growth and metastasis of tumor, including extracellular matrix and stromal cells (fibroblasts, vasculature cells and inflammatory cells). CiberSort algorithm was used to evaluate the relationship between risk score and immune cells (Supplementary Table S7). It was found that risk score was negatively correlated with naive B cells, resting dendritic cells, CD8 + T cells, follicular helper T cells and regulatory T cells. Positive correlations were found with resting memory CD4 + T cells, M0 macrophages, activated mast cells, and neutrophils (Fig. 8A). In addition, the relationship between the expression of two candidate genes and the abundance of immune cells was evaluated, and it was found that these two genes affect most immune cells (Fig. 8B). The organ chart showed that the tumor microenvironment scores (including immune scores, matrix scores and estimated scores) of the high-risk group were all higher than those of the low-risk group (Fig. 8C). The distribution of somatic mutations was different in high and low risk groups. TMB in low-risk group was higher than that in high-risk group. The waterfall diagram visualizes the 20 genes with the highest mutation frequency in the high and low risk groups, and TP53 is the gene with the highest mutation frequency (Fig. 8D, E), suggesting that there is a potential correlation between cuproptosis in lung cancer cells and tumor mutations. The scatter plot showed a negative correlation between risk scores and tumor stem cells (R = -0.5, *P* < 0.001), indicating that LUSC cells with higher cell retention gene scores exhibited more stem cell characteristics and less differentiation (Fig. 8F).

### Chemotherapy sensitivity

**Fig. 9:**
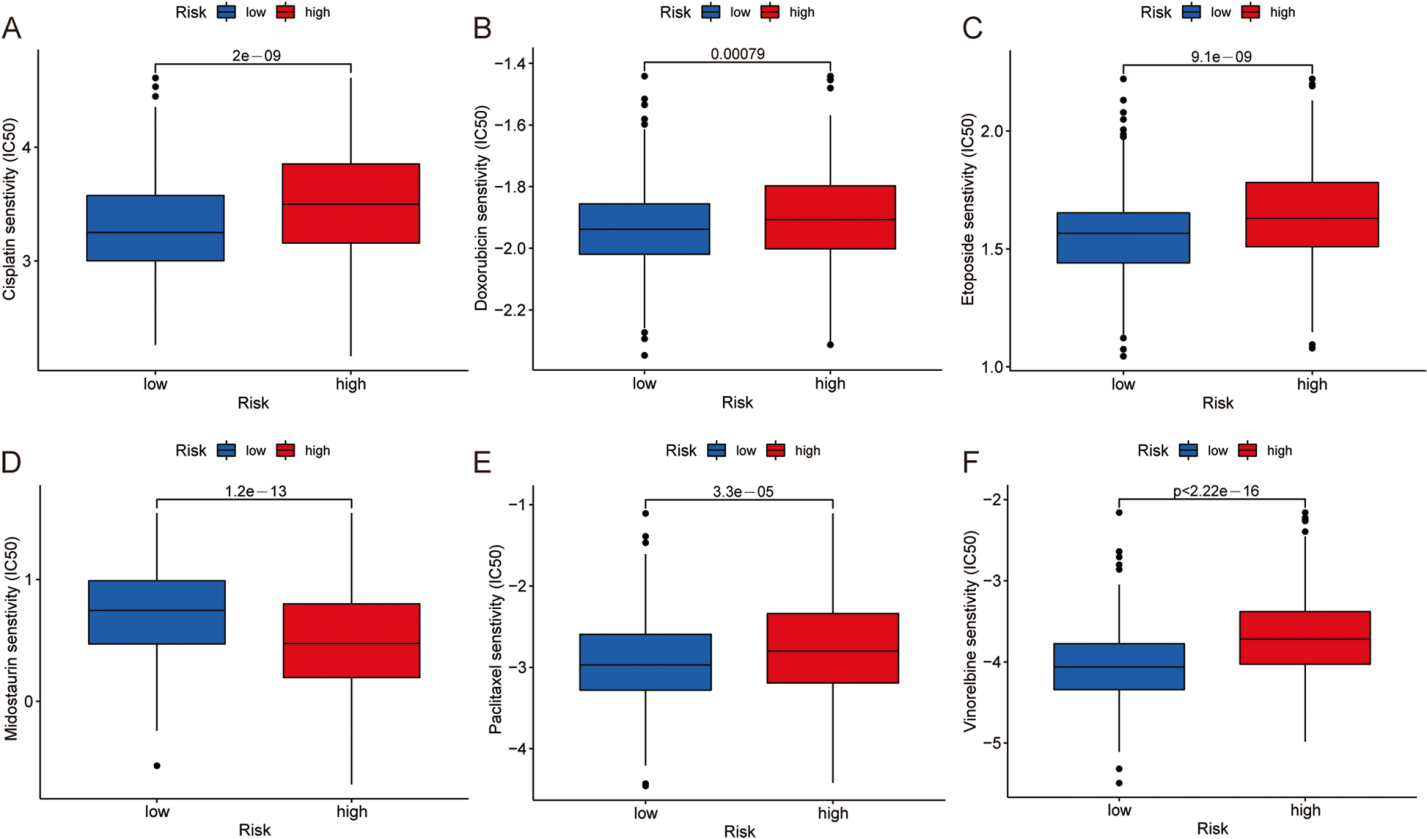
Sensitivity analysis of chemotherapy drugs. **A**-**F** IC50 values of cisplatin, doxorubicin, etoposide, paclitaxel, vinorelbine and middotolin

The sensitivity analysis of several chemotherapeutic agents used in the treatment of LUSC was performed in the two groups. IC50 values of cisplatin, doxorubicin, etoposide, paclitaxel, vinorelbine and other drugs in high-risk group were higher, while IC50 values of middoxoline and other drugs in low-risk group were higher (Fig. 9A-F). It is clear from these results that CRG are essential for drug sensitivity, which means that these drugs have a potential role in the future treatment of LUSC.

## Discussion

Although patients with LUSC have made progress in surgery, chemotherapy, radiotherapy and immunotherapy, the prognosis of patients with advanced or metastatic LUSC is still poor. Therefore, the discovery of new diagnostic tools is crucial for evaluating cancer prognosis.

When imbalance of copper homeostasis in human body induces the death of tumor cells through a variety of different mechanisms, including copper-induced oxidative stress, inhibition of ubiquitin–proteasome system, copper deficiency inhibiting angiogenesis of tumor cells, and cuproptosis found in recent studies [30]. Cuproptosis is a form of copper-dependent cell death, is related to the TCA cycle in mitochondria. Copper ions directly bind to the acylated components in the tricarboxylic acid cycle pathway, resulting in abnormal aggregation of acylated proteins and loss of iron-sulfur cluster proteins, triggering a protein toxic stress response and ultimately mediating cell death [31]. Copper, like all metallic elements, is divided into two parts within the cell: the tightly bound protein part and the bioavailable ion state part [32]. Mitochondria are important organelles for storing copper. In mitochondria, cytochrome coxidase assembly factor 6 (COA6) and cytochrome oxidase 2 (SCO2) help to maintain the redox balance of cytochrome oxidase 1 (SCO1), which binds to copper and passes it to cytochrome coxidase ( COX), thereby activating the enzyme activity in the respiratory chain [33]. Compared with normal cells, cancer cells have a higher demand for copper; some cancers express a large number of lipoic acylated mitochondrial proteins and exhibit high-intensity respiration. In our study, according to GSVA enrichment analysis, subtype A of cuproptosis model was more abundant than subtype B in metabolic activation pathway. Kaplan–Meier survival curve analysis showed that the survival prognosis of subtype B was better than that of subtype A, and there was significant difference (P = 0.011). This is consistent with previous studies. At present, the relevant mechanism research of cuproptosis and tumors should be further deepened. Although many bioinformatics studies have explored the significance of cuproptosis in the clinical diagnosis and prognosis of various tumors [34–37]. However, there is no research on the role of CRGs in LUSC. Therefore, it is of great practical significance to classify LUSC patients according to CRGs and construct a more accurate prognostic model.

In this study, we investigated the effect of CRGs on survival and prognosis in patients with LUSC. In the TCGA cohort, apart from *LIPT1, DBT,* and *DLST*, we found that the expressions of other 16 CRGs among the 19 were significantly different in LUSC tissues and normal tissues. The expression levels of 10 CRGs were significantly correlated with the prognosis of patients with LUSC (*p* < 0.05). First of all, based on the expression of CRGs, patients were divided into two different molecular subtypes (Cluster A and Cluster B). Survival analysis showed that the prognosis of patients with subtype B was significantly better than that of subtype A. In order to explore the potential mechanism of prognostic differences between the two subtypes, GO function enrichment analysis and KEGG pathway enrichment analysis were performed. It was found that most of these gene functions were related to immune regulation and cell adhesion. There were also significant differences in the degree of immune cell infiltration between the two CRGs molecular subtypes. Twenty-three immune cells were selected for analysis. Except for CD56 dim natural killer cell and CD56 bright natural killer cell, the infiltration degree of the other 21 immune cells in the two subtypes was significantly different, and the infiltration level of immune cells in subtype B was lower than that in subtype A.

Then 736 differentially expressed genes were screened by single factor cox analysis, which were expressed in both cuproptosis subtypes. Three gene subtypes (A, B, C) were identified based on DEGs between the two cuproptosis subtypes. Subtype C had the best survival outcome and subtype A had the worst survival outcome. Then prognostic genes were screened from 736 DEGs. After 336 prognostic related genes were obtained, two key variable genes (*FSTL3* and *RGMA*) were selected by LASSO algorithm for model construction and verification. Studies have found that *FSTL3* can promote the metastasis of tumor cells by forming an inhibitory immune microenvironment [38, 39], and promote the polarization of macrophages and fibroblasts [40]. *RGMA* acts as a tumor suppressor gene in colorectal cancer, breast cancer, prostate cancer, lymphoma and other tumors [41–43]. In a study of colorectal cancer, it was found that overexpression of *RGMA* inhibited the proliferation, migration and invasion of tumor cells, and increased cell apoptosis, which can be used as an important marker for early diagnosis and prognosis of tumors [44].

Then LUSC patients were divided into high and low risk groups according to the median risk score. A more accurate nomogram was constructed by combining clinical parameters and risk scores. The calibration curve shows that the actual results are in good agreement with the predicted results. Survival analysis found that overall survival at high-risk group was significantly lower compared to the low-risk group across all samples, training sets, and test sets. ROC and C-index curve were used to verify the prognostic accuracy of risk score. We found that the risk score can be used as a criterion for predicting prognosis. In addition, our study found that there was a correlation between risk score and the number of immune cells. The immune score, matrix score and estimated score of the high-risk group were higher than those of the low-risk group, and the TMB of the low-risk group was higher than that of the high-risk group. It can be seen that the overall survival time of patients in the high-risk group with a high tumor microenvironment score is shorter than that in the low-risk group with a low tumor microenvironment score. Suggesting that the content of matrix components and immune components in the tumor microenvironment can be used as an indicator to judge the prognosis of patients with LUSC.

The tumor microenvironment is a complex environment for tumor cells to survive, including extracellular matrix and stromal cells, including fibroblasts, vasculature cells (epithelial cells, outer membrane and smooth muscle cells) and immune cells (lymphocytes, macrophages, dendritic cells, mast cells and neutrophils), which play an important role in tumor growth, invasion and metastasis. Immune cells play a key role in the occurrence and development of tumors, and also have an important impact on the treatment outcome and prognosis of patients [45]. The tumor microenvironment is characterized by ion homeostasis imbalance, acidic environment, hypoxia, increased lactic acid, decreased glucose concentration, nutritional competition, and changes in the secretory group, which can lead to metabolic reprogramming of immune cells and change their proper functions. So as to show reduced inflammatory response or enhanced inhibitory function, thus assisting tumor immune escape. In turn, it strengthens the resistance of tumor cells to immunotherapy, promotes the overexpression of immune checkpoint molecules and tumor metastasis [46–48]. Based on these characteristics, immunotherapy has become a new direction in tumor therapy. Immune checkpoint is a class of immunosuppressive molecules that can regulate the body's immune response. In the process of the occurrence and development of tumor, immune checkpoint immune tolerance is an important mechanism to acquired immune escape, and immune checkpoint inhibitors (ICIs) can inhibit tumor of inhibitory signals, enhance immune cell tumor killing activity. In recent years, a number of clinical studies have shown that compared with traditional chemotherapy, immune checkpoint inhibitors alone or in combination with chemotherapy can significantly improve the efficacy and prognosis of patients with advanced lung squamous cell carcinoma, and even cause changes in the treatment mode of patients with lung squamous cell carcinoma [24, 49, 50].

In our study, we noted that the low-risk group had a higher TMB. TMB is the total number of substitutions and insertions / deletions per megabase in the exon coding region of the evaluated gene in a tumor sample [51]. Studies have shown that tumors with a higher mutation load can recruit more neoantigens on the surface of tumor cells, increase the immunogenicity of tumors, and improve the efficacy of immunotherapy [52]. Tumor gene mutations are highly specific, with different gene mutation profiles among different patients. TMB may be a potential biomarker for immunotherapy outcomes of multiple solid tumors. In recent years, more and more research data show that TMB is a novel biomarker of immune checkpoint inhibitors, which can predict the efficacy of immunotherapy [53].

Finally, we also analyzed the drug susceptibility of high-risk group and low-risk group, and screened 86 drugs with significant differences in IC50 concentration between high-risk group and low-risk group. Among them, it is worth paying attention to cisplatin, doxorubicin, etoposide, paclitaxel, vinorelbine, middoxoline, etc.

In this study, a prognostic model was constructed based on the expression of CRGs. The results showed that the model had high accuracy in predicting the prognosis of LUSC patients. Differences in immune status and TMB between high-risk and low-risk groups suggest that CRGs may affect the immune response of LUSC and further affect the prognosis of patients with LUSC. Finally, sensitivity analysis of chemotherapy drugs also provided new directions for anticancer drug discovery and may help in the development of individualized treatment programs.

Unavoidably, the study has certain limitations. First, the study was based on secondary analyses of public databases and sample size was small. These retrospective data were subject to selection bias, which affected the accuracy of the analysis results. Thus, the CRGs prognostic signature needs to be further verified with prospective, multicenter, and clinical data. Second, further functional experiments need to be performed to investigate the potential molecular mechanisms of CRGs in the role of immune microenvironment and tumor progression of LUSC. Third, given the nature of the study, it is impossible to access all known prognostic factors and further studies are needed. Finally, the function of potential drugs targeting the prognostic signature needs further in-depth studies.

## Conclusion

In summary, this study constructed a risk prediction model based on CRGs and showed good predictive effect, which can be used to predict the prognosis of LUSC. According to the risk score, the differences of tumor microenvironment, immune status and chemosensitivity between different risk score groups were revealed. It provides a valuable basis for further research on the diagnosis or individualized treatment of LUSC patients, and guides individualized treatment including immunotherapy and chemotherapy.

## Supplementary Information

**Additional file 1: Table S1.** The list of cuproptosis-related genes. **Table S2.** The correlation between cuproptosis-related genes and OS in LUSC patients. **Table S3.** The list of 1233 DEGs. **Table S4.** Risk Score of train group. **Table S5.** Risk Score of test group. **Table S6.** Genes identified by Cox regression. **Table S7.** The relationship between the CRG score and the number of immune cells.

## Data Availability

The datasets used and analyzed during the current study are available within the manuscript and its figures and tables.

## Abbreviations

LUSC: Lung squamous cell carcinoma
CRG: Cuproptosis-related gene
TCGA: The Cancer Genome Atlas
GEO: Gene Expression Omnibus
NSCLC: Non-small cell lung cancer
LUAD: Lung adenocarcinoma
EGFR: Epidermal growth factor receptor
ALK: Anaplastic lymphoma kinase
TCA: Tricarboxylic acid cycle
DEG: Differentially expressed genes
